# Enhancing Cancer Patient Navigation: Lessons from an Evaluation of Navigation Services in Alberta, Canada

**DOI:** 10.3390/curroncol32050287

**Published:** 2025-05-21

**Authors:** Linda Watson, Se’era May Anstruther, Claire Link, Siwei Qi, Kathryn Burrows, Michelle Lack, Krista Rawson, Andrea DeIure

**Affiliations:** 1 Cancer Care Alberta, Alberta Health Services, Calgary, AB T2N 5G2, Canadaclaire.link@albertahealthservices.ca (C.L.); siwei.qi@albertahealthservices.ca (S.Q.); kathryn.burrows@albertahealthservices.ca (K.B.); michelle.lack@albertahealthservices.ca (M.L.); krista.rawson@albertahealthservices.ca (K.R.); andrea.deiure@albertahealthservices.ca (A.D.); 2 Faculty of Nursing, University of Calgary, Calgary, AB T2N 1N4, Canada

**Keywords:** cancer patient navigation, oncology, nursing, equity intervention, ambulatory oncology, person-centered care delivery

## Abstract

Cancer patient navigation has emerged as a patient-centric intervention enabling equitable cancer care, by mitigating barriers patients encounter throughout their cancer journey. Cancer Care Alberta (CCA) implemented a professional navigation model over a decade ago and commissioned a program evaluation in response to evolving operational demands. The objectives were (1) to better understand the current state of CCA’s cancer patient navigation program; (2) to explore the need for other specialized streams; and (3) to provide key recommendations to strengthen and grow the program. A mixed methods approach, including a survey, administrative data, and semi-structured interviews, captured patient-, staff-, and system-level insights. Findings revealed difficulties in identifying complex patients needing navigation, along with inconsistencies regarding intake practices, program awareness, referral pathways, standardized workflows, and a lack of programmatic supports, which contributed to variability in service delivery. A need for enhanced palliative navigation support also emerged. Approximately 25% of surveyed patients reported being unable to access perceived needed support before their first oncology consultation. These findings underscore the importance of early, targeted navigation for equity-deserving populations. Recommendations include harmonizing program structure, refining navigator roles, expanding navigation streams, standardizing processes, and enhancing equity-focused competencies. These findings offer a roadmap with which to improve person-centered cancer care.

## 1. Introduction

Cancer patient navigation has emerged as a patient-centric intervention that enables equitable cancer care, helping to mitigate the complexities and barriers patients with cancer encounter throughout their healthcare journey [1]. The definition of cancer patient navigation has evolved over time, but can be defined as “the role and activities that enable people affected by cancer to overcome healthcare barriers and facilitate access to quality health and psychosocial care across the cancer care continuum” [2]. Navigation programs were developed to address the inequities in cancer outcomes related to factors such as socioeconomic status, race/ethnicity, geographic location, and communication as well as information barriers [2,3,4].

In Alberta, Canada, oncology care is embedded within the publicly funded provincial healthcare system. Cancer Care Alberta (CCA) delivers ambulatory oncology services through a network of 17 sites across the province. CCA provides care and support for adult patients living with cancer, starting from diagnosis through to survivorship or palliative care [5]. Person-centered care is a core approach within CCA that includes the principles of respect and dignity, information sharing, patient and family participation, and collaboration to improve care services [6]. This approach has demonstrated improvements in patient experience, quality of care, staff performance, and overall health outcomes [7,8]. To enhance person-centered cancer care in Alberta, patient navigation emerged as a key strategy to better support patients as they move through the complex cancer system.

In 2006, a provincial needs assessment was conducted to identify barriers and inequities in cancer care across Alberta [9]. The assessment identified two primary needs: (1) improve linkages between patients and families, community care agencies, and healthcare providers in primary, ambulatory, and acute care settings across the entire cancer care trajectory, and (2) increase navigation supports in rural and isolated settings, where access and continuity of care were more challenging. To address these needs, CCA adopted a professional navigation model using oncology-trained registered nurses, capable of supporting patients at any point in the cancer care trajectory. This approach was especially important in rural areas, where oncology expertise and supportive care resources are more limited [10,11]. A pilot project conducted in two rural community cancer centers and one isolated urban regional cancer center between 2008 and 2010 demonstrated positive outcomes, leading to recommendations for program expansion. With funding from the Alberta Cancer Foundation, CCA implemented a provincial navigation model in 2012 within all 15 rural and isolated urban settings. Over time, the cancer patient navigation model has evolved to include different specialized navigation streams, including Adolescent and Young Adult (AYA) (ages 18–39), Indigenous Cancer Patient Navigation (ICPN), Rural, and Regional Navigation.

Within CCA, regardless of the specialized stream, Cancer Patient Navigators (CPNs) serve as a central point of contact for patients, families, and healthcare providers seeking information about cancer care, bridging clinical care and supportive services across the cancer care continuum [12,13]. Their responsibilities include facilitating access to supportive care resources, coordinating care, delivering health education, and improving communication between patients and the healthcare system [14]. Over time, the role of CPNs at regional sites evolved to incorporate intake triage to meet operational requirements for support with new patient consultations. As a result, Regional Navigators have a blended role that includes triaging new referrals to the first consultation as well as offering person-centric navigation supports as required. In contrast to the blended Regional Navigator role, the other CPNs (AYA, Indigenous, and Rural) do not engage in new referral triage and focus specifically on supporting distinct patient populations experiencing barriers to care, who have complex care coordination needs.

In 2022, CCA’s navigation program reached a key milestone of ten years in operation. However, program staff were struggling to keep up with growing demands for care. Furthermore, the program had no metrics for CPN staffing growth related to increasing patient volumes, complexity, or need. As the volume of patients requiring CPN care grew, further inequities in access to navigation were occurring, largely related to the lack of a consistent strategy to identify which patients would benefit from CPN support. In response, CCA leadership commissioned a comprehensive programmatic evaluation of the cancer patient navigation program. The objectives of this evaluation were (1) to better understand the current state of CCA’s cancer patient navigation program; (2) to explore the need for additional specialized streams; and (3) to provide key recommendations to strengthen and grow the program. This article reports on the program evaluation and findings. These findings may offer valuable insights for other provincial or comparable cancer patient navigation programs seeking to enhance patient-centered care, address health inequities, and improve system efficiency. By sharing lessons from this evaluation, the authors aim to contribute to the continued development and refinement of navigation services to better meet the needs of patients living with cancer.

## 2. Materials and Methods

### 2.1. Study Design and Population

This evaluation employed mixed methods to thoroughly explore CCA’s cancer patient navigation program. Quantitative measures included administrative data analysis and a patient experience survey, while qualitative data were gathered through semi-structured interviews to gain deeper insights into the program’s processes and workflows, and experiences of being a CPN. Both qualitative and quantitative data were synthesized and interpreted simultaneously. As multiple data sources were utilized, the sample size, time frame, and analysis varied based on the data source.

### 2.2. Administrative Data

To understand the activity within the navigation program, administrative data on patients cared for by a CPN were retrospectively gathered from all 17 CCA sites between 7 November 2022 and 6 November 2023. This time frame was selected as CCA implemented a new program-wide electronic medical record on 6 November 2022; therefore, this was the first full year of navigation data that could be accessed. These data included details about the individuals who received CPN care, including their type of cancer, where they lived, what navigator services they received, and other demographic information. Visit data were also included, highlighting the number and frequency of CPN visits the patient received.

Patient-Reported Outcomes (PROs) are routinely collected across all CCA sites. Data from all patients who completed at least one PRO measure between 7 November 2022 and 6 November 2023 were included in the administrative analyses. This allowed a comparison of symptom burden between the patients who received CPN care and patients who did not. The validated PRO measure used in CCA is the revised Edmonton Symptom Assessment System for Cancer (ESAS-r Cancer) [15]. The ESAS-r Cancer assesses the severity of 15 symptoms from 0 to 10 (10 being the most severe), including pain, tiredness, drowsiness, nausea, lack of appetite, shortness of breath, depression, anxiety, wellbeing, diarrhea, constipation, numbness or tingling, sleep problems, thinking problems, and mobility problems. The mean scores of all symptoms reported were used to compare the symptom burden in navigated and unnavigated cohorts. These comparisons were descriptive and not subjected to formal statistical testing.

### 2.3. Ambulatory Oncology Patient Satisfaction Survey

The Ambulatory Patient Satisfaction Survey (AOPSS) is a validated cancer patient experience survey that is distributed biennially to patients who have received ambulatory treatments within CCA [9]. The AOPSS is administered to a random sample of patients in the province who are currently undergoing cancer treatment or who have been treated within the past six months to understand areas of strength and identify areas for improvement. As the survey was distributed while this evaluation was in progress (February 2023), a pragmatic decision was made to add a new question to further inform the evaluation. The following question was added to the survey: “While you were waiting for your first appointment with the cancer program, which of the following supports did you need and could not access? Please select all that apply: (1) information about treatments for your cancer; (2) access to someone who could answer your questions and help navigate the cancer system; (3) mental health supports (such as those for depression or anxiety); (4) practical supports (such as financial aid); (5) social supports (such as contact with family or friends); and (6) other”.

### 2.4. Semi-Structured Interviews

Three key stakeholder groups participated in the semi-structured interviews: leaders from other provincial cancer patient navigation programs across Canada; operational managers within CCA who managed CPNs as part of their clinical teams; and CPNs themselves. To assess programmatic structures and supports that facilitate effective CPN programs, interviews were conducted with provincial leaders within three provincial CPN programs, including Cancer Care Manitoba (CCMB), the Nova Scotia cancer program, and within cancer programs in Quebec. Participants were recruited via email invitations, with a total of 122 individuals consenting to participate in audio-recorded interviews. Interviews were conducted between April and October 2023. All interview guides included questions about general topics such as referral processes, training for CPNs, key clinical activities within the program, documentation, workflows, operational supports and challenges, and patient care. Multiple interview guides were created to tailor questions to the specific group being interviewed. To address the core objectives of the evaluation, each guide was structured to explore participants’ experiences with navigation delivery, perceptions of current program gaps, and suggestions for how to improve the program. Four evaluation team members were involved in conducting interviews, although not all members of the team were in every interview. Interviews were conducted in English, recorded online using Microsoft Teams, and ranged from 20 min to 2 h.

### 2.5. Data Analysis

Quantitative data were analyzed using SPSS (version 29; IBM Corp., Armonk, NY, USA) and SAS Enterprise (version 9.4; SAS Institute Inc., Cary, NC, USA). Descriptive statistics were used to summarize cancer patient navigation program characteristics, patient demographic data, and survey data.

Qualitative data from the interviews were transcribed verbatim. Four team members employed thematic analysis to identify the main categories [16]. Any disagreements were discussed among team members until a consensus was reached. Strategies to enhance qualitative rigor included methodological, data, and investigator triangulation [17,18]. Direct quotes from participants were included to maintain credibility, ensuring the accurate reflection of participants’ perspectives [19].

### 2.6. Ethical Considerations

This project complies with the Helsinki Declaration and the Alberta Research Ethics Community Consensus Initiative (ARECCI) guidelines for quality improvement and evaluation [20]. A project screen established by the ARECCI identified that the current project did not require full board review as it determined this was a quality improvement project. No harm was anticipated or reported concerning this project.

## 3. Results

### 3.1. Program Metrics and Patient-Reported Outcomes

A total of 6203 unique patients engaged in at least one CPN encounter across CCA between 7 November 2022 and 6 November 2023. An individual patient may engage with multiple navigation streams; thus, the sum of the sample size values presented for each cohort is greater than the number of unique patients. In the AYA, Indigenous, and Rural streams, more patients were female. AYA patients were more likely to reside in the two largest metropolitan areas of the province (75.9%) and be diagnosed with hematological cancer (26%). Patients cared for by ICPN navigators were more likely to be in the 40–64-year age group (49.4%), reside in a rural area (52.8%), and be diagnosed with gastrointestinal cancer (23.1%). Patients in the Rural (49.3%) and Regional (65.5%) navigation streams were more likely to be 65 years and older. Table 1 presents additional characteristics of the administrative data sample.

Of the unique patients who had at least one CPN encounter across CCA, a combined total of 20,040 CPN encounters were recorded (Table 2). These interactions included direct contact and indirect care coordination. The average number of CPN encounters per patient engaged was 3.23. Approximately 2322 patients (37.4% of the total number of patients) had one CPN encounter, and 1459 (23.5%) had two CPN encounters within the time range. CPN encounters per patient ranged from 1 encounter to a maximum of 63 encounters. When categorized by navigation stream, the ICPN stream exhibited the highest average per-person rate at 8.16.

Table 3 presents unique patients and total encounters by demographic and clinical characteristics such as age group, sex, and tumor group, revealing variations in the utilization of navigation services among different demographic groups. Notably, patients aged between 40 and 64 exhibited the highest average CPN encounters. Female patients demonstrated a slightly higher average of CPN encounters than males.

**Figure 1:**
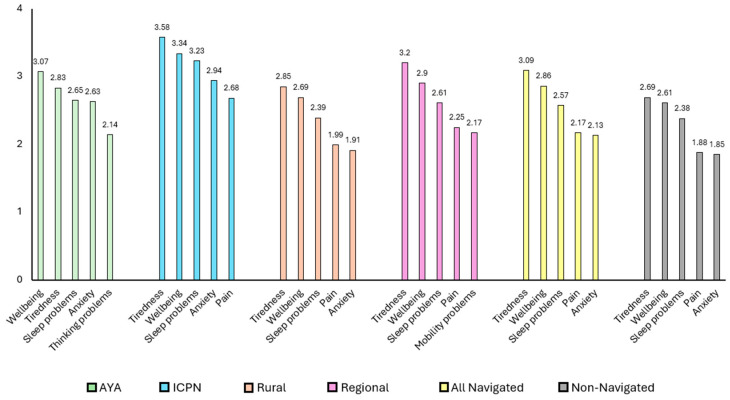
Top five severe symptoms across cohorts.

In order to understand the symptom burden between navigated and non-navigated patients, PRO data from 42,077 CCA patients were analyzed in two cohorts: those that received CPN care and had completed a PRO questionnaire in the one-year time frame (*n =* 4357 unique patients) and those who had completed a PRO questionnaire, but had not received CPN care in that time frame (*n =* 37,720). The data showed that navigated patients reported a substantially higher symptom burden compared to those who did not receive navigation (Figure 1). This suggests that CCA staff may be referring more symptomatic patients to CPNs. However, there is no standard process to determine which patients should be referred to CPN care.

Further analysis of PRO data indicated that symptoms and severity varied across cohorts of navigated patients. As seen in Figure 1, AYA patients reported wellbeing, tiredness, sleeping problems, anxiety, and thinking problems as their most severe symptoms, with higher scores in wellbeing, anxiety, and thinking problems than the non-navigated group. Indigenous patients reported their top five most severe symptoms as tiredness, wellbeing, sleeping problems, anxiety, and pain, scoring higher than both the navigated and non-navigated groups across these symptoms. While rural patients demonstrated generally lower symptom severity among specialty cohorts, their tiredness and pain scores remained relatively higher than those of the provincial cancer population. Lastly, the Regional group highlighted their top five severe symptoms as tiredness, wellbeing, sleeping problems, pain, and mobility problems, and reported higher scores in those five symptoms than the non-navigated group.

### 3.2. Improving Patient Experiences of Care: Earlier CPN Care as a Strategy

**Figure 2:**
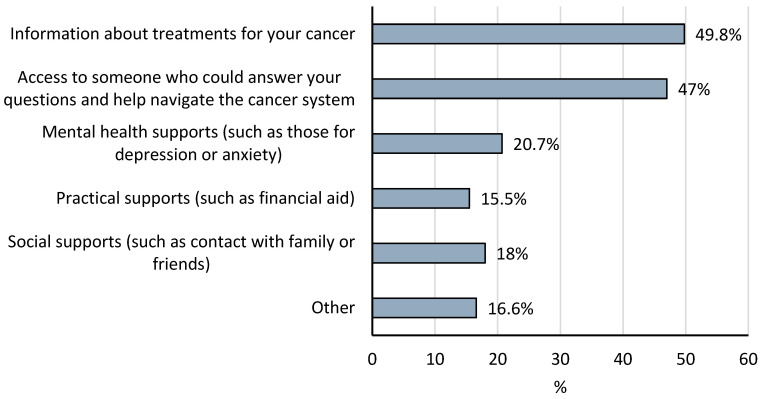
Types of support wanted by patients who felt they could not access support.

Of the respondents who completed the AOPSS question (*n* = 1798), three-quarters of patients indicated they could access all the support they needed or did not need any support after they were referred to CCA but before their first consultation with the oncology team. However, approximately one-quarter (*n* = 440) of the respondents felt they needed at least one support during this early time frame but could not access it. As it is not financially feasible to have every CCA patient assigned a CPN, this finding is helpful as it begins to quantify a basic denominator regarding the subset of patients who may benefit from CPN care. Figure 2 presents the different types of support respondents felt they needed. Information about cancer treatment (49.8%), along with access to someone to answer questions and help navigate the cancer system (47%), was wanted by almost half of respondents. Secondary analysis of the AOPSS data revealed that this subset of patients also had lower satisfaction across care domains while in CCA’s care. Table 4 presents the sociodemographic information of this sub-group. Compared with the full survey sample, patients in need of support were more likely to be younger, female, reside in large urban centers, have less social support, and report poorer health. Currently in CCA, there is no standardized approach at new patient referral or other time points for identifying patients who are experiencing barriers to accessing care, making it difficult to identify patients who may benefit from early navigation interventions.

### 3.3. The Challenges of Being a CPN

Across interviews with the specialty CPNs, a clear sense emerged that even after ten years, there remains a significant lack of awareness about the program from both patient and organizational perspectives. Referrals to navigators are ad hoc, which limits the ability to connect with patients early in their cancer journey. One navigator reflected that “We need to be more efficient at getting our information out to patients that lets them know we are support available to them”. Too often I catch people later in their journey and patients say, “I wish I would have known about you sooner”. Relatedly, navigators also consistently shared the importance of connecting with patients early in their care trajectory. One Regional CPN shared that “The navigator role is very valuable to the patient, especially at the start of the patient’s journey. Knowing the process, understanding what and why things are happening, having some basic information about what to expect, knowing there is someone to reach out to for questions, concerns, and help, that makes a real difference”. However, due to high patient volumes and competing workloads, Regional CPNs shared that it was difficult to navigate patients beyond the initial intake phase, making it especially difficult to best support patients with an advanced cancer diagnosis who require more specialized support compared to patients diagnosed at earlier stages.

All CPNs felt some degree of moral distress related to their inability to provide navigation support to some patients due to changes in clinic workload. They also described feeling that their work is undervalued by managers and other clinical team members. This sentiment was most strongly expressed by Rural navigators, who were often reassigned to meet broader clinical needs, such as covering staff shortages in other departments, without corresponding adjustments to their navigation workload. One participant shared that “Our role is not always understood by staff in CCA, which leads to collaboration and communication challenges and lost opportunities to help patients earlier in their trajectory”.

Navigators in all specialty streams echoed concerns about limited recognition and the emotional toll of managing highly complex cases, often involving patients facing significant barriers to care or advancing illness. For example, when a new referral is received for a patient who has advanced (late-stage) cancer at diagnosis, there is little difference in how support is provided to these patients prior to their first consultation. Therefore, CPNs are left to find their own way to meet the needs of late-stage patients. The problem solving required in each case can be exhausting, and feeling that the work is undervalued can lead to burnout. CPNs expressed the need for greater support in managing the emotional demands of the role and emphasized the value of stronger collaboration with supportive care teams, particularly given the clinical depth and disease-specific knowledge their work requires.

### 3.4. More Programmatic Support Is Needed

Participants also highlighted several areas where greater programmatic support was needed. Many noted a lack of clarity around role expectations and limited access to professional practice support to address complex care situations. Navigators frequently work in isolation, as the sole representative of their role at a given site, leading to uncertainty around best practices and workload management. Administrative tasks consume a large portion of their time and participants voiced the need for clerical support, structured vacation coverage, and cross-training to ensure service continuity. They also emphasized the importance of ongoing training to better serve equity-deserving populations, such as newcomers, LGBTQ2S+ individuals, older adults, and those experiencing housing instability. Finally, given their involvement with patients with advanced disease, specialty CPNs identified a pressing need for further education and support in delivering palliative care. All specialty navigators are often involved in supporting patients with advanced cancer, and the level of support these patients require tends to increase as their disease worsens.

### 3.5. Programmatic Structures and Supports for Other Cancer Patient Navigation Programs

In the interviews with provincial leaders of cancer patient navigation programs, many similarities were identified regarding the challenging nature of the CPN role and the need for programmatic support for role development, training, and standard referral pathways. The cancer navigation programs in Nova Scotia and Quebec closely resembled Alberta’s program, with their focus on supporting coordination and access to care for patients receiving ambulatory oncology care. However, CCMB’s navigation program differed in that it focused on supporting patients in the pre-diagnostic work-up phase until they are referred into the Provincial Cancer Program. Both Nova Scotia and Quebec leaders described current work underway to develop additional navigation capacity to support cancer patients prior to their first ambulatory oncology consultation, as this pre-consult phase was recognized as a particularly difficult time for cancer patients.

## 4. Discussion

This evaluation explored the current state of CCA’s cancer patient navigation program, the need for expanded specialized navigation streams, and potential recommendations with which to strengthen the program. The findings reinforce the essential role of CPNs in coordinating care, facilitating access to supportive services, and the added potential of addressing patient needs early in the cancer care trajectory. While the program has evolved from a primarily rural-based CPN program to include Regional, AYA, and Indigenous navigation, several systemic challenges persist, including a limited presence of intake processes aimed at identifying patients who may have challenges accessing equitable care, insufficient awareness within CCA of the CPN program, unclear referral pathways to CPN care, and a lack of standardized workflows and programmatic supports (i.e., CPN role clarity and competency development). These gaps contribute to variability in service delivery and limit the ability of navigators to consistently support patients experiencing barriers to care. To strengthen the programmatic nature of the CPN intervention, a recommendation was made to have all CPNs report to a single manager to create more opportunity for consistent practice supports, workflows, metrics, and capacity building within the program [21].

The regional navigation role blends navigation and triage, primarily supporting intake triage (referral to first oncology consultation). However, the original CPN role was conceptualized to also support patients requiring complex navigation support later in their care trajectory. Due to the high volume of new referrals at regional sites and the time-sensitive nature of triage work, there is currently limited capacity for Regional CPNs to address the navigation needs of patients beyond the initial intake phase, especially at high-volume regional centers. To reduce the moral distress of CPNs in this blended role, a recommendation was made to limit the scope of Regional CPN roles to focus on a patient’s triage and navigation needs prior to the first consultation. If the patient requires ongoing navigation support, added capacity for referral to specialty CPN care should be explored at Regional sites.

There is a need for additional navigation streams to improve patient experiences and better support CPNs in their role. Based on internal data, 3200 new referrals are received annually by CCA for patients diagnosed with advanced (late-stage) cancer [22]. Additionally, there is an increasing national trend towards patients with non-curative cancer living longer due to ongoing treatments for control [23]. Within CCA, AOPSS data from 2021 indicated that approximately half of all respondents reported that their treatment intent was to control rather than cure their cancer [24]. Such data paired with the interview feedback from CPNs demonstrate the increased need for palliative care and related navigation support. As a result, efforts are already underway at CCA to develop a new Early Palliative Navigation stream. This program is currently being piloted, with initial feedback indicating a positive impact. The specialty navigation program will require standardized workflows for referrals, consistent documentation practices, and the reporting of key metrics to support ongoing quality improvement [2].

Higher symptom burden scores observed among navigated patients suggest that navigation services appropriately target individuals experiencing greater symptom burden and complexity. However, the evaluation revealed notable gaps in systematically identifying patients requiring greater support during the initial referral-to-consultation period. Approximately one in four patients felt unsupported during this phase, highlighting a significant opportunity to improve patient care. Evidence suggests that certain factors are associated with an increased need for support following a cancer diagnosis, including racialized groups, where incidence rates may be similar, but mortality rates are higher across cancer types [25]; being very young or very old [26,27]; limited social supports; geographic isolation in rural settings [28]; economic barriers [29,30]; the presence of co-morbid conditions [31]; low health literacy [32]; and communication difficulties with healthcare teams [33,34]. Despite these known risk factors, CCA does not routinely collect this type of information. Implementing a provincial standardized intake screen that captures symptom burden, social determinants of health, and informational needs could proactively identify patients who would benefit from additional navigation support, potentially improving patient satisfaction and outcomes [35,36]. Furthermore, navigation programs fundamentally serve as equity interventions. Ensuring further competency development specific to supporting equity-deserving groups, including Indigenous populations, immigrants, LGBTQ2S+, geriatric patients, and individuals experiencing housing instability, will enable nurses to deliver culturally safe, inclusive, and comprehensive care.

### Limitations and Future Directions

There are several limitations to the evaluation that should be considered. First, while our findings may offer valuable guidance, they are specific to CCA’s organizational structure and may not be directly generalizable to other jurisdictions with different healthcare structures, patient populations, or navigation models. Second, CCA does not currently collect detailed sociodemographic and social determinants of health data during patient intake, which limited the ability to systematically identify and analyze needs of equity-deserving populations. However, work is currently underway to develop an equity-informed screening tool soon after a patient has been referred to CCA, creating a consistent approach to identifying patients experiencing complexity that would benefit from care from a CPN. Lastly, interview data are subject to some degree of social desirability bias. To help mitigate this limitation, interviews were conducted by a team member who held no supervisory authority over participants to reduce the likelihood of power dynamics influencing responses.

Despite these limitations, the findings of this evaluation have substantially influenced ongoing developments within CCA’s cancer patient navigation program. CCA has implemented a harmonized CPN program management strategy with all CPNs reporting to a common manager. The new manager will strive to support unified workflows, promote consistent CPN role activities through standardized orientation and programmatic professional development activities, develop key performance indicators for the CPN program, and develop a staffing model that will guide program growth. As discussed, work within the program has begun to add an early palliative stream of CPN care. As the program evolves, and as new patient care needs/populations emerge, there may be a need for other navigation streams to be developed. There should be further evaluation of specialty streams to assess their impact on patient outcomes and experience, care coordination, and equity in accessing supportive care. Moreover, given the limited literature on nurse-led cancer patient navigation programs in Canada, additional studies are necessary to expand the evidence base and inform best practices across diverse cancer care settings.

## 5. Conclusions

Cancer patient navigation programs are a critical nursing-led strategy for overcoming barriers and fostering equitable access to comprehensive cancer care. While CCA’s cancer patient navigation program evolved over the past decade to address emerging patient needs through the addition of different navigation streams, this evaluation identified opportunities with which to enhance care further by addressing gaps in program standardization, decreasing variability in care processes and creating the capacity to deliver navigation services in a consistent and coordinated manner. As a result of this evaluation, actionable insights are now informing changes to the program’s structure, processes, and focus areas. These recommendations and actions are guided by a desire to support a more integrated, proactive, and equitable navigation program that is better positioned to meet the complex needs of patients living with cancer across the province. Collectively, these findings provide a practical roadmap for provincial cancer navigation programs aiming to enhance person-centered care and optimize system-wide efficiencies.

## Figures and Tables

**Table 1:** Sociodemographic and clinical characteristics of patients by navigation stream *.

Characteristic	AYA (*n* = 373)		Indigenous (*n* = 360)		Rural (*n* = 1829)		Regional (*n* = 3862)	
	N	%	N	%	N	%	N	%
Age Group								
<=17	1	0.3	2	0.6	8	0.4	0	0.0
18–39	333	89.3	47	13.1	120	6.6	138	3.6
40–64	38	10.2	178	49.4	799	43.7	1195	30.9
>=65	1	0.3	133	36.9	902	49.3	2529	65.5
Sex								
Female	236	63.3	210	58.3	1036	56.6	1908	49.4
Male	137	36.7	149	41.4	793	43.4	1954	50.6
Unknown	0	0.0	1	0.3	0	0.0	0	0.0
Rurality								
Metro	283	75.9	107	29.7	233	12.7	67	1.7
Urban	56	15.0	29	8.1	468	25.6	2106	54.5
Rural	10	2.7	190	52.8	1104	60.4	1634	42.3
Unknown	24	6.4	34	9.4	24	1.3	55	1.4
Tumor								
BR	88	23.6	78	21.7	394	21.5	866	22.4
CNS	30	8.0	10	2.8	25	1.4	17	0.4
Endocrine	9	2.4	10	2.8	22	1.2	21	0.5
GI	28	7.5	83	23.1	363	19.8	703	18.2
GU	20	5.4	52	14.4	222	12.1	865	22.4
GYNE	20	5.4	37	10.3	133	7.3	111	2.9
H&N	4	1.1	16	4.4	49	2.7	49	1.3
HEM	97	26.0	26	7.2	257	14.1	328	8.5
LNG	6	1.6	26	7.2	192	10.5	528	13.7
Melanoma	5	1.3	4	1.1	33	1.8	59	1.5
Non-melanoma skin	0	0.0	3	0.8	12	0.7	54	1.4
Other malignant	14	3.8	12	3.3	98	5.4	226	5.9
Sarcoma	52	13.9	3	0.8	29	1.6	35	0.9

* The sum of the sample size values presented for each cohort is greater than the number of unique patients due to some patients engaging with multiple navigation streams. Abbreviations: BR: Breast; CNS: Central Nervous System; GI: Gastrointestinal; GU: Genitourinary; GYNE: Gynecological; H&N: Head & Neck; HEM: Hematology; LNG: Lung.

**Table 2:** Number of patients, encounters, and CPN encounters per patient by navigation stream.

Navigation Stream	Number of Patients	Number of Encounters	Average CPN Encounters per Patient
Regional	3862	9424	2.44
Rural	1829	6966	3.81
ICPN	360	2937	8.16
AYA	373	713	1.91
Total	6203 *	20,040	3.23

* Number of unique patients. The sum of the values presented for each cohort is greater than the number of unique patients due to some patients engaging with multiple navigation streams.

**Table 3:** Cancer patient navigation encounters by patient characteristics.

Characteristic	Unique Patients		Encounters		Average Encounters
	*n* = 6203	%	*n* = 20,040	%	
Age Group					
<=17	9	0.1%	21	0.1%	2.33
18–39 *	584	9.4%	1927	9.6%	3.30
40–64	2116	34.1%	7718	38.5%	3.65
65+	3494	56.3%	10,374	51.8%	2.97
Sex					
Female	3264	52.6%	10,972	54.8%	3.36
Male	2938	47.4%	9066	45.2%	3.09
Unknown	1	0.0%	2	0.0%	2.00
Tumor Group					
BR	1381	22.3%	4413	22.0%	3.20
GI	1120	18.1%	4110	20.5%	3.67
GU	1124	18.1%	3017	15.1%	2.68
GYNE	289	4.7%	1066	5.3%	3.69
HEM	686	11.1%	2138	10.7%	3.12
LNG	732	11.8%	2584	12.9%	3.53
OTHER **	871	14.0%	2712	13.5%	3.11

* The demographic of individuals aged 18–39 recorded 1927 CPN encounters, contrasting with the 713 documented AYA encounters. This is because patients within the 18–39 age range could have overlapped with other navigation streams (ICPN, Rural, and/or Regional); ** Other tumour groups include CNS, endocrine, H&N, melanoma, non-melanoma skin, other malignant, and sarcoma. Abbreviations: BR: Breast; CNS: Central Nervous System; GI: Gastrointestinal; GU: Genitourinary; GYNE: Gynecological; H&N: Head & Neck; HEM: Hematology; LNG: Lung.

**Table 4:** Sociodemographic characteristics of patients who felt they could not access support.

Characteristic	Needed Support and Could Not Access It (*n* = 440)	Full Provincial Sample (*n* = 1939)
Age *M* (SD)	66.1 (12.5)	68.3 (11.3)
Age Groups		
18–39	13 (3.1%)	33 (1.8%)
40–54	60 (14.5%)	166 (9.1%)
55–64	105 (25.3%)	399 (21.9%)
65–74	119 (28.7%)	669 (36.7%)
75–84	96 (23.1%)	454 (24.9%)
85+	22 (5.3%)	100 (5.5%)
Sex		
Female	247 (59.5%)	962 (52.8%)
Male	168 (40.5%)	859 (47.2%)
Median Household Income (Neighborhood-Level)		
CAD < 50,000	19 (4.6%)	89 (4.9%)
CAD 50,000–<75,000	105 (25.4%)	487 (26.8%)
CAD 75,000–<100,000	98 (23.7%)	491 (27.1%)
CAD 100,000–<125,000	112 (27.1%)	412 (22.7%)
CAD 125,000 or more	79 (19.1%)	335 (18.5%)
Rurality of Residence		
Metro	229 (55.2%)	888 (48.8%)
Urban	65 (15.7%)	393 (21.6%)
Rural	121 (29.2%)	540 (29.7%)
Treatment Facility		
Tertiary	274 (62.3%)	1119 (57.7%)
Regional	117 (26.6%)	577 (29.8%)
Community	49 (11.1%)	243 (12.5%)
Education		
High school or less	147 (34.4%)	656 (36.2%)
Some college or university	74 (17.3%)	264 (14.6%)
College, trade, or technical degree	106 (24.8%)	467 (25.8%)
University degree (bachelor’s or higher)	100 (23.4%)	426 (23.5%)
Self-Rated Health		
Excellent/very good	139 (32.6%)	637 (35.1%)
Good	166 (38.9%)	786 (43.3%)
Fair/poor	122 (28.6%)	393 (21.6%)
Social Support ^1^		
Yes, completely	332 (79.2%)	1566 (87.2%)
Somewhat/no	87 (20.8%)	230 (12.8%)
Chronic Conditions Before Cancer		
None	142 (34.5%)	615 (35.3%)
At least one	270 (65.5%)	1126 (64.7%)
Goal of Cancer Treatment ^2^		
To cure my cancer	196 (47.9%)	896 (50.7%)
To control my symptoms	199 (48.7%)	839 (47.5%)
I don’t know	14 (3.4%)	33 (1.9%)

^1^ “Overall, do you feel you have enough support from a partner, family member, or friend?”; ^2^ “What was the goal of your treatment?”.

## Data Availability

The data used in this study are not publicly available; however, they may be available upon request with appropriate ethical and operational approval.

## References

[1] Dixit N., Rugo H., Burke N.J. (2021). Navigating a Path to Equity in Cancer Care: The Role of Patient Navigation. Am. Soc. Clin. Oncol. Educ. Book.

[2] Chan R.J., Milch V.E., Crawford-Williams F., Agbejule O.A., Joseph R., Johal J., Dick N., Wallen M.P., Ratcliffe J., Agarwal A. (2023). Patient Navigation Across the Cancer Care Continuum: An Overview of Systematic Reviews and Emerging Literature. CA Cancer J. Clin..

[3] Rivers B.M., Rivers D.A. (2022). Enhancing Cancer Care Coordination Among Rural Residents: A Model to Overcome Disparities in Treatment. Med. Care.

[4] Freeman H.P., Rodriguez R.L. (2011). History and Principles of Patient Navigation. Cancer.

[5] Future of Cancer Impact (FOCI) in Alberta. https://www.albertahealthservices.ca/scns/page14126.aspx.

[6] Alberta Health Services Patient & Family Centred Care in Action. https://www.albertahealthservices.ca/info/Page16748.aspx.

[7] The Health Foundation Person-Centred Care Made Simple What Everyone Should Know About Person-Centred Care. https://www.health.org.uk/sites/default/files/PersonCentredCareMadeSimple.pdf.

[8] Ebrahimi Z., Patel H., Wijk H., Ekman I., Olaya-Contreras P. (2021). A Systematic Review on Implementation of Person-Centered Care Interventions for Older People in out-of-Hospital Settings. Geriatr. Nurs..

[9] Miller J. (2006). (The Praxis Group, Edmonton, AB, Canada). Internal document.

[10] Cantril C., Haylock P.J. (2013). Patient Navigation in the Oncology Care Setting. Semin. Oncol. Nurs..

[11] Champ S., Dixon C. (2024). Cancer Patient Navigation in Canada: Directions from the North. Semin. Oncol. Nurs..

[12] Watson L.C., Anderson J., Champ S., Vimy K., Delure A. (2016). Developing a Provincial Cancer Patient Navigation Program Utilizing a Quality Improvement Approach Part Two: Developing a Navigation Education Framework. Can. Oncol. Nurs. J..

[13] Katerenchuk J., Salas A.S. (2023). An Integrative Review on the Oncology Nurse Navigator Role in the Canadian Context. Can. Oncol. Nurs. J..

[14] Watson L.C., Vimy K., Anderson J., Champ S., DeIure A. (2016). Developing a Provincial Cancer Patient Navigation Program Utilizing a Quality Improvement Approach. Part Three: Evaluation and Outcomes. Can. Oncol. Nurs. J..

[15] Watson L., Qi S., Link C., DeIure A., Afzal A., Barbera L. (2023). Patient-Reported Symptom Complexity and Acute Care Utilization Among Patients with Cancer: A Population-Based Study Using a Novel Symptom Complexity Algorithm and Observational Data. J. Natl. Compr. Cancer Netw..

[16] Braun V., Clarke V. (2006). Using Thematic Analysis in Psychology. Qual. Res. Psychol..

[17] Patton M.Q. (1999). Enhancing the Quality and Credibility of Qualitative Analysis. Health Serv. Res..

[18] Ahmed S.K. (2024). The Pillars of Trustworthiness in Qualitative Research. J. Med. Surg. Public Health.

[19] Slevin E., Sines D. (2000). Enhancing the Truthfulness, Consistency and Transferability of a Qualitative Study: Utilising a Manifold of Approaches. Nurse Res..

[20] Hagen B., O’Beirne M., Desai S., Stingl M., Pachnowski C.A., Hayward S. (2007). Innovations in the Ethical Review of Health-Related Quality Improvement and Research: The Alberta Research Ethics Community Consensus Initiative (ARECCI). Healthc. Policy.

[21] Pratt-Chapman M.L., Silber R., Tang J., Le P.T.D. (2021). Implementation Factors for Patient Navigation Program Success: A Qualitative Study. Implement. Sci. Commun..

[22] Cancer Care Alberta (2023). (Alberta Health Services, Edmonton, AB, Canada). Internal document.

[23] Pituskin E. (2022). Cancer as a New Chronic Disease: Oncology Nursing in the 21st Century. Can. Oncol. Nurs. J..

[24] Watson L., Link C., Qi S., DeIure A. (2023). Quantifying the Impact of Family Doctors on the Care Experiences of Patients with Cancer: Exploring Evidence from the 2021 Ambulatory Oncology Patient Satisfaction Survey in Alberta, Canada. Curr. Oncol..

[25] Pinheiro L.C., Reshetnyak E., Akinyemiju T., Phillips E., Safford M.M. (2022). Social Determinants of Health and Cancer Mortality in the Reasons for Geographic and Racial Differences in Stroke (REGARDS) Cohort Study. Cancer.

[26] Berben L., Floris G., Wildiers H., Hatse S. (2021). Cancer and Aging: Two Tightly Interconnected Biological Processes. Cancers.

[27] Alvarez E.M., Force L.M., Xu R., Compton K., Lu D., Henrikson H.J., Kocarnik J.M., Harvey J.D., Pennini A., Dean F.E. (2022). The Global Burden of Adolescent and Young Adult Cancer in 2019: A Systematic Analysis for the Global Burden of Disease Study 2019. Lancet Oncol..

[28] Lung Cancer and Equity Report: A Focus on Income and Geography. https://www.partnershipagainstcancer.ca/topics/lung-cancer-equity/social-determinants-health/#:~:text=Without%20regular%20access%20to%20health,at%20greater%20risk%20of%20cancer.

[29] Ward E., Jemal A., Cokkinides V., Singh G.K., Cardinez C., Ghafoor A., Thun M. (2004). Cancer Disparities by Race/Ethnicity and Socioeconomic Status. CA Cancer J. Clin..

[30] Clegg L.X., Reichman M.E., Miller B.A., Hankey B.F., Singh G.K., Lin Y.D., Goodman M.T., Lynch C.F., Schwartz S.M., Chen V.W. (2009). Impact of Socioeconomic Status on Cancer Incidence and Stage at Diagnosis: Selected Findings from the Surveillance, Epidemiology, and End Results: National Longitudinal Mortality Study. Cancer Causes Control.

[31] Sarfati D., Koczwara B., Jackson C. (2016). The Impact of Comorbidity on Cancer and Its Treatment. CA Cancer J. Clin..

[32] Holden C.E., Wheelwright S., Harle A., Wagland R. (2021). The Role of Health Literacy in Cancer Care: A Mixed Studies Systematic Review. PLoS ONE.

[33] Hendren S., Chin N., Fisher S., Winters P., Griggs J., Mohile S., Fiscella K. (2011). Patients’ Barriers to Receipt of Cancer Care, and Factors Associated with Needing More Assistance from a Patient Navigator. J. Natl. Med. Assoc..

[34] Bourgeois A., Horrill T.C., Mollison A., Lambert L.K., Stajduhar K.I. (2023). Barriers to Cancer Treatment and Care for People Experiencing Structural Vulnerability: A Secondary Analysis of Ethnographic Data. Int. J. Equity Health.

[35] Jarelnape A.A., Ali Z.T., Fadlala A.A., Sagiron E.I., Osman A.M., Abdelazeem E., Balola H., Albagawi B. (2023). The Influence of Nursing Interventions on Patient Outcomes: A Systematic Review. Saudi J. Health Syst. Res..

[36] Novilla M.L.B., Goates M.C., Leffler T., Novilla N.K.B., Wu C.-Y., Dall A., Hansen C. (2023). Integrating Social Care into Healthcare: A Review on Applying the Social Determinants of Health in Clinical Settings. Int. J. Environ. Res. Public Health.

